# A Challenging Case of Biliary Obstruction in an 82-Year-Old Male With a History of Zollinger-Ellison Syndrome and Suspected Cholangiocarcinoma Secondary to Biliary Candidiasis

**DOI:** 10.7759/cureus.77551

**Published:** 2025-01-16

**Authors:** Muhammad Waqar Elahi, Mona Ghias, Asif Haris, David Shi

**Affiliations:** 1 Internal Medicine, West Virginia University, Morgantown, USA

**Keywords:** biliary candidiasis, biliary obstruction, candidiasis, cholangitis, ercp, fungal biliary infection, s: cholangiocarcinoma, zollinger-ellison syndrome

## Abstract

Biliary obstruction is a serious condition with various underlying causes, including malignancy, infection, and gallstones. Fungal biliary infections are rare, frequently misdiagnosed, and carry significant morbidity and mortality. Here, we present the case of an 82-year-old male with multiple comorbidities who developed sepsis secondary to cholangitis. Despite initial diagnostic challenges and the complexity of his biliary anatomy due to prior surgeries, a multidisciplinary approach identified biliary candidiasis as the underlying cause of cholangitis and sepsis. This case underscores the importance of considering fungal infections in the differential diagnosis of biliary obstruction, particularly in high-risk and immunosuppressed patients. Early recognition is essential to enable prompt treatment and improve patient outcomes.

## Introduction

Biliary obstruction, a blockage of the bile ducts, can lead to serious complications such as cholangitis (infection of the bile ducts), jaundice (yellowing of the skin and eyes), and liver damage. While cholangiocarcinoma, a cancer of the bile ducts, is a well-recognized cause of biliary obstruction, fungal infections of the biliary tree are exceedingly rare. Biliary obstruction caused by fungal infection is particularly uncommon and sparsely documented in the literature [1]. Most reported cases of biliary candidiasis have occurred in patients with underlying malignancies, diabetes mellitus, or other immunosuppressive conditions, such as cystic fibrosis [1]. Biliary candidiasis should prompt suspicion for biliary or regional malignancy, necessitating a thorough workup to confirm or rule out such diagnoses and facilitate timely treatment [1]. Here, we present the case of an 82-year-old male with multiple comorbidities who exhibited signs and symptoms of sepsis secondary to cholangitis. Further evaluation revealed biliary candidiasis as the underlying cause of his cholangitis.

## Case presentation

The patient was an 82-year-old male with a complex medical history, including major depressive disorder, coronary artery disease, hypertension, cerebrovascular accident, Zollinger-Ellison syndrome status post tumor resection, Roux-en-Y gastrojejunostomy, Alzheimer’s dementia, and benign prostatic hyperplasia. He presented to the emergency room with altered mental status after nursing home staff noted increased confusion compared to his baseline. Upon presentation, the patient was alert and oriented only to person. His blood pressure was low at 86/59 mmHg, while other vital signs were within normal limits.

Significant laboratory values are presented in Table 1.

**Table 1:** Laboratory data

Parameter	Result	Reference value
White blood cell count	14.1 × 10⁹/L	3.7-11 × 10⁹/L
C-reactive protein	117.6 mg/L	≤8.0 mg/L
Procalcitonin, serum	3.24 ng/mL	<0.5 ng/mL
Bilirubin, total	2.9 mg/dL	0.3-1.3 mg/dL
Bilirubin, conjugated	1.1 mg/dL	<0.3 mg/dL
Aspartate aminotransferase (SGOT)	160 U/L	8-48 U/L
Alanine transaminase (SGPT)	154 U/L	<55 U/L
Alkaline phosphatase	340 U/L	45-115 U/L
Creatinine	1.67 mg/dL	0.62-1.27 mg/dL
Blood urea nitrogen	29 mg/dL	8-25 mg/dL

**Figure 1:**
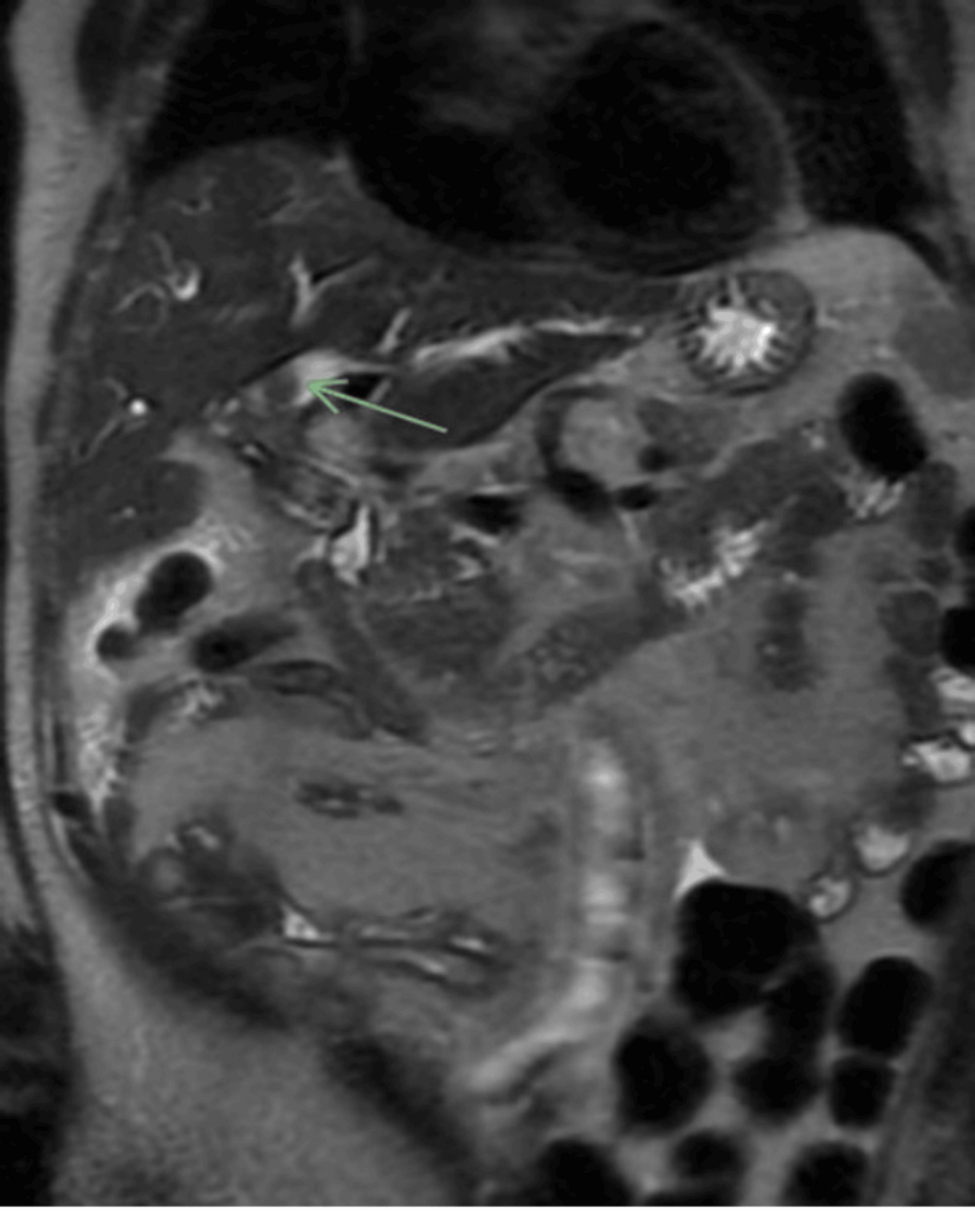
Intraluminal filling defect within the intrahepatic ducts observed on a coronal SSFSE sequence SSFSE, single-shot fast spin-echo

**Figure 2:**
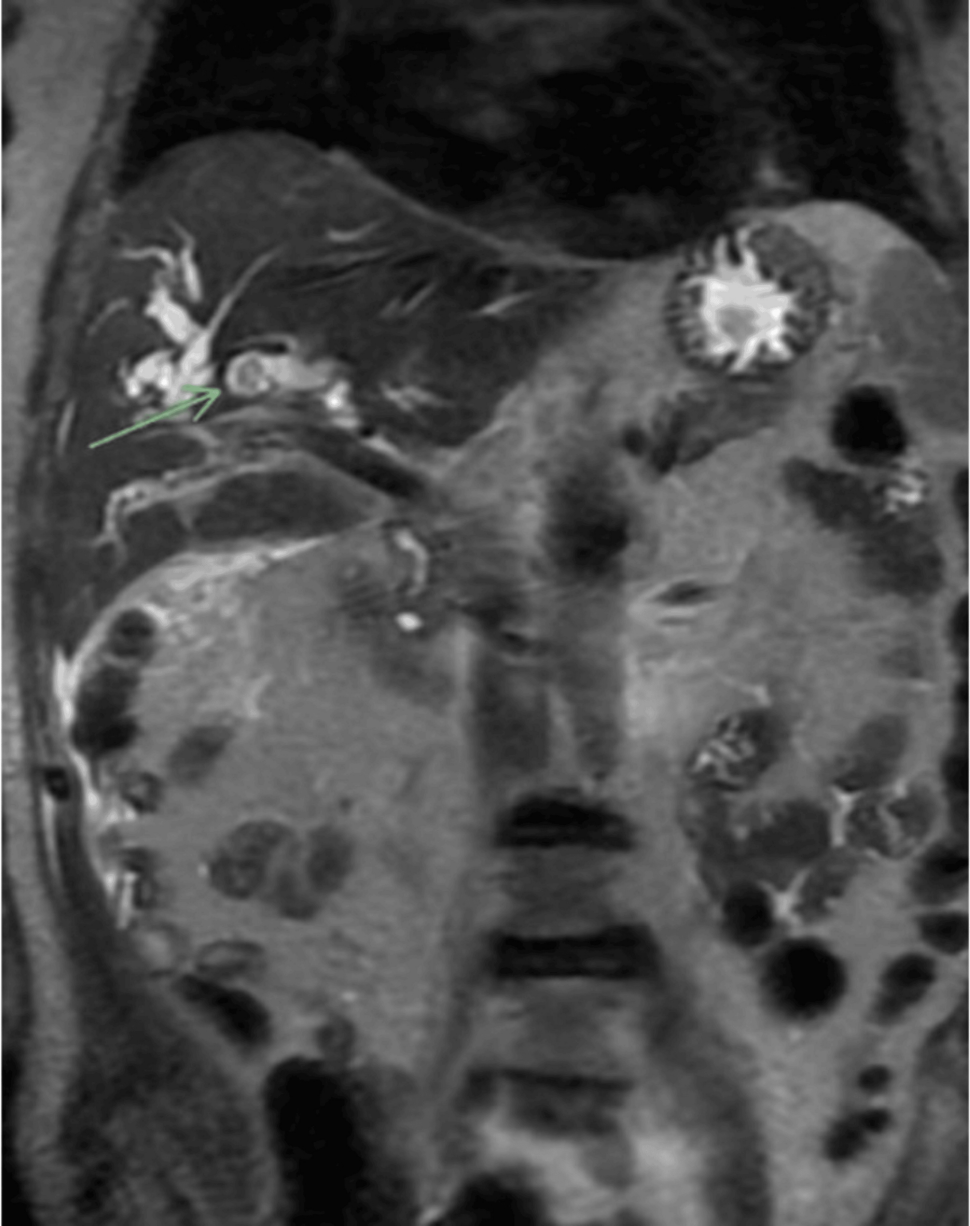
Suggestion of an additional intraluminal filling defect within the intrahepatic ducts on coronal SSFSE sequence SSFSE, single-shot fast spin-echo

The patient underwent a magnetic resonance cholangiopancreatography, which revealed marked intrahepatic biliary ductal dilatation with a central filling defect, highly concerning for cholangiocarcinoma (Figure 1, Figure 2).

**Figure 3:**
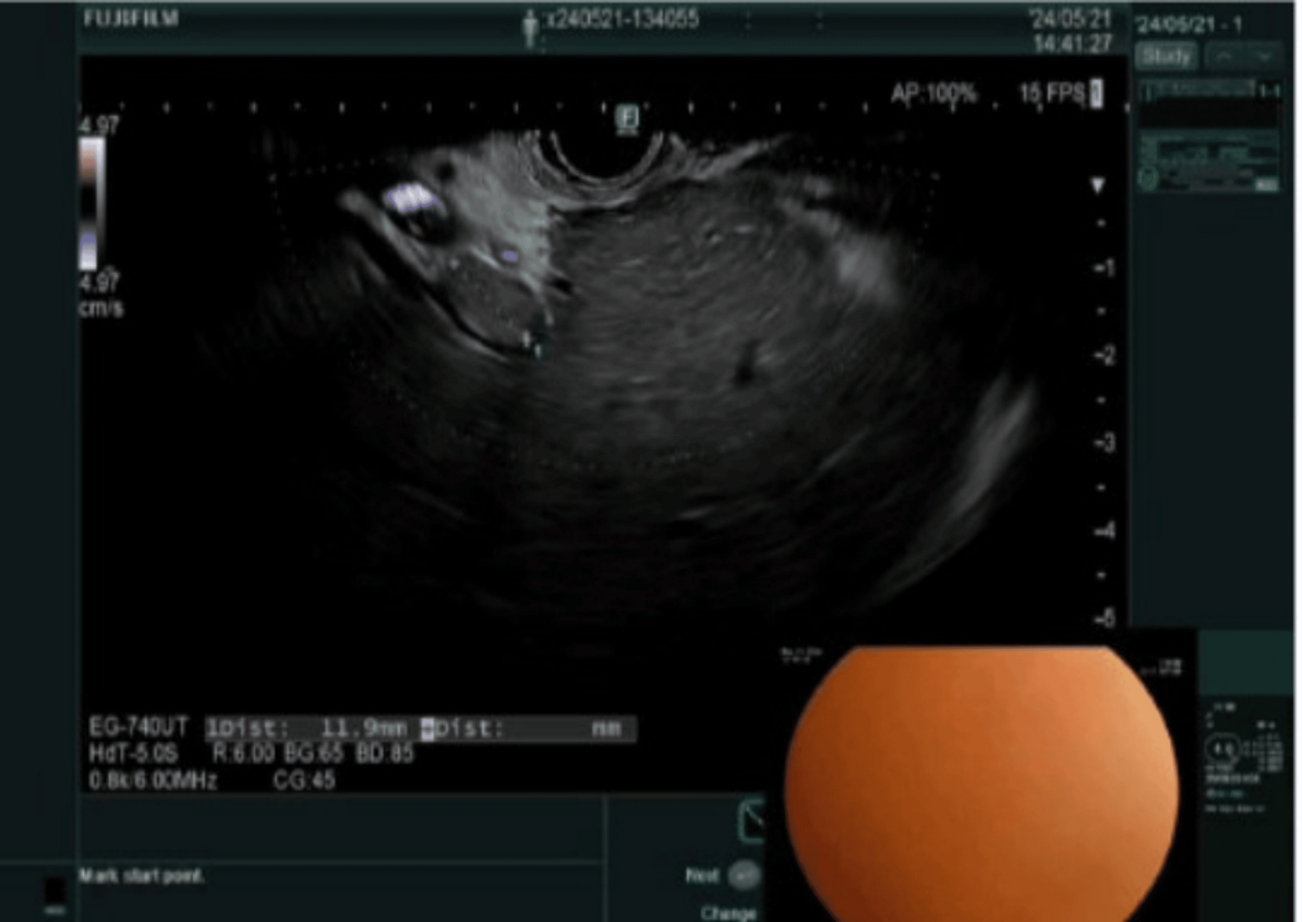
Intraductal sludge

**Figure 4:**
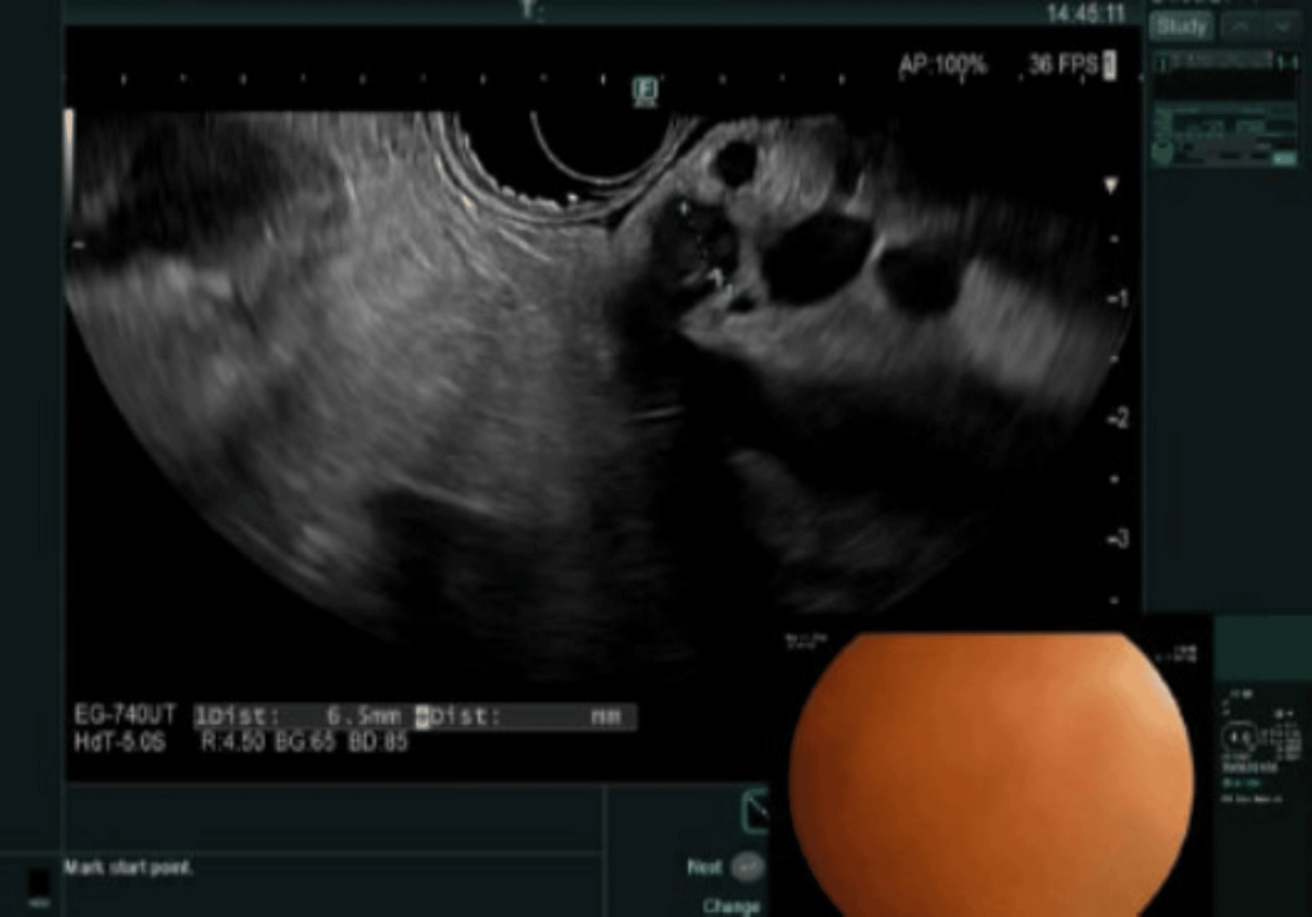
Proximal CBD CBD, common bile duct

The patient was found to be in sepsis, likely secondary to cholangitis. Blood cultures grew *Escherichia coli*, and he was started on ertapenem. Gastroenterology was consulted. On the fifth day of hospitalization, the patient underwent endoscopic ultrasound (EUS) and endoscopic retrograde cholangiopancreatography (ERCP), but the procedure was aborted due to difficult cannulation related to his prior Roux-en-Y hepaticojejunostomy. EUS revealed intrahepatic biliary ductal dilation with intraductal sludge (Figure 3) and an apparent surgical transection of the proximal common bile duct (CBD; Figure 4).

**Figure 5:**
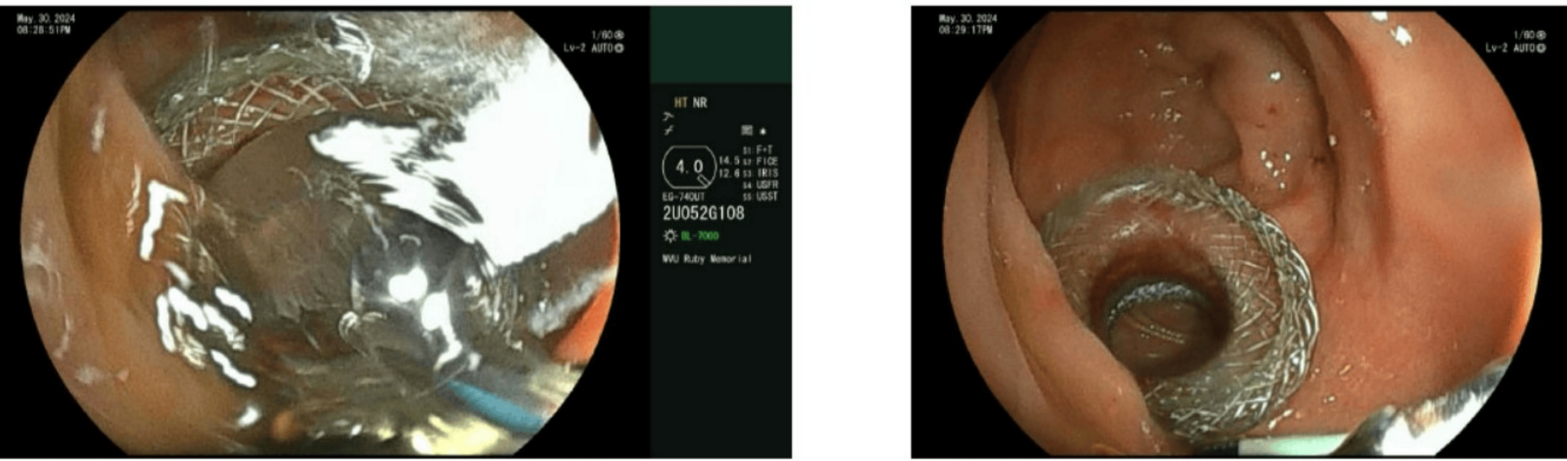
LAMS with dilation LAMS, lumen-apposing metal stent

A multidisciplinary conference was held, and the decision was made to proceed with the creation of a gastrojejunostomy using a lumen-apposing metal stent (LAMS) to facilitate subsequent EUS-directed transenteric ERCP. After a few days, the advanced endoscopy team successfully created the gastrojejunostomy using LAMS, and the stent was dilated to 18 mm (Figure 5).

A week later, EUS-directed transenteric ERCP was performed, which revealed a single localized biliary stricture in the lower third of the main bile duct, along with choledocholithiasis, which was completely removed by balloon extraction. Cytology brushing at the site of the stricture was negative for malignancy but showed fungal organisms morphologically consistent with *Candida* species. Biliary candidiasis was treated with a 14-day course of fluconazole.

## Discussion

Biliary obstruction and cholangitis are common clinical issues, with bacterial infections being the typical cause. However, in recent years, involvement of the biliary tract by *Candida *and other fungal species has been increasingly reported [2]. “Biliary candidiasis” refers to yeast infection within the biliary tract, although cases remain limited to a few reports and case series.

The clinical manifestations of *Candida *infection can range from localized disease to widespread, disseminated infections with multisystem organ failure. *Candida albicans* is the most common species implicated, although other species can also cause severe infections [3]. Fungal infections have also been associated with CBD obstruction [4,5].

Risk factors for biliary candidiasis include immunosuppression, diabetes, prolonged antibiotic therapy, extended critical care stays, ERCP [6], and the use of external biliary shunts/endoprostheses [7]. Other independent risk factors for intra-abdominal candidiasis include intra-abdominal surgeries, gut perforation, anastomotic leakage, and the presence of an abdominal drain [8].

Due to challenges in obtaining bile samples, our knowledge of bile duct microbial flora remains limited. Moreover, it is unclear whether positive fungal findings represent true infection or simple colonization, as *Candida* is considered part of the normal microbiota in the gastrointestinal and genitourinary tracts of humans [3]. A prospective observational study by Lenz et al. [2] concluded that positive fungal cultures are not mere contamination artifacts, especially in patients with significant risk factors. The study also suggested that previous biliary tract manipulation may increase the risk of biliary tract infections [1,9]. Treatment typically involves bile duct drainage and antifungal therapy.

This case underscores the complexities of managing biliary obstruction, particularly in patients with confounding medical histories and altered biliary anatomy. Biliary candidiasis can closely mimic cholangiocarcinoma, complicating the diagnostic process. This case highlights the importance of considering both possibilities, especially in patients with risk factors for fungal infections. Furthermore, EUS-guided techniques, such as gastrojejunostomy creation, can be valuable tools in overcoming anatomical challenges and achieving successful biliary intervention.

## Conclusions

This case illustrates the successful application of a multidisciplinary approach, including EUS with gastrojejunostomy creation followed by transenteric ERCP, to manage a challenging biliary obstruction in a patient with a complex medical history. The patient was septic and diagnosed with biliary candidiasis. This case emphasizes the importance of considering biliary candidiasis as a potential cause of biliary obstruction, particularly in high-risk patients.
